# A fast algorithm for the multiple genome rearrangement problem with weighted reversals and transpositions

**DOI:** 10.1186/1471-2105-9-516

**Published:** 2008-12-04

**Authors:** Martin Bader, Mohamed I Abouelhoda, Enno Ohlebusch

**Affiliations:** 1 Institute of Theoretical Computer Science, University of Ulm, 89069 Ulm, Germany; 2 Faculty of Engineering, Cairo University, Giza, Egypt; 3 Nile University, Giza, Egypt

## Abstract

**Background:**

Due to recent progress in genome sequencing, more and more data for phylogenetic reconstruction based on rearrangement distances between genomes become available. However, this phylogenetic reconstruction is a very challenging task. For the most simple distance measures (the breakpoint distance and the reversal distance), the problem is NP-hard even if one considers only three genomes.

**Results:**

In this paper, we present a new heuristic algorithm that directly constructs a phylogenetic tree w.r.t. the weighted reversal and transposition distance. Experimental results on previously published datasets show that constructing phylogenetic trees in this way results in better trees than constructing the trees w.r.t. the reversal distance, and recalculating the weight of the trees with the weighted reversal and transposition distance. An implementation of the algorithm can be obtained from the authors.

**Conclusion:**

The possibility of creating phylogenetic trees directly w.r.t. the weighted reversal and transposition distance results in biologically more realistic scenarios. Our algorithm can solve today's most challenging biological datasets in a reasonable amount of time.

## Background

During evolution both local and global mutations of DNA molecules occur. Local mutations (point mutations) consist of the substitution, insertion, or deletion of single nucleotides, while global mutations (genome rearrangements) change the DNA molecules on a large scale. In unichromosomal genomes, the most common rearrangements are inversions (also called reversals in bioinformatics), where a section of the genome is excised, reversed in orientation, and re-inserted. But also transpositions play a role. In a transposition, a section of the genome is excised and inserted at a new position in the genome; this may or may not also involve an inversion. Since genome rearrangements are rare compared to point mutations, they can give us valuable information about the evolutionary history of organisms. Moreover, because of the progress in large-scale sequencing in the last decade, hundreds of complete genomes are available to date. As a consequence, we are now able to tackle the problem of reconstructing the evolutionary history of genomes.

In the context of genome rearrangements, a unichromosomal genome is usually represented by an ordering of certain oriented markers (e.g., genes or synteny blocks [1]). Moreover, in the comparison of several genomes, it is typically assumed that the genomes have the same set {1,...,*n*} of markers. Thus, in the following a unichromosomal genome will be represented by a signed permutation of the sequence (1,...,*n*), where the sign indicates the corresponding orientation (strandedness) of the marker.

In the multiple genome rearrangement problem, one searches for a phylogenetic tree describing the most "plausible" rearrangement scenario for multiple genomes. Formally, given *k *genomes, find a tree *T *with the *k *genomes as leaf nodes and assign ancestral genomes to internal nodes of *T *such that the tree is optimal, i.e., the sum of rearrangement distances over all edges of the tree is minimal. The following rearrangement distances have been studied:

• The *breakpoint distance *between two genomes is the smallest number of places where one genome must be broken so that the pieces can be rearranged to form the other genome. It can straightforwardly be computed in linear time.

• The *reversal distance *between two genomes is the minimum number of reversals required to transform one genome into the other. It can also be computed in linear time [2,3].

• In the *weighted reversal and transposition distance*, the two types of operations are assigned (different) weights, and the rearrangement distance between two genomes is the minimum of the weights of all rearrangement sequences that transform one genome into the other (of course, the weight of a sequence is the sum of the weights of the operations in the sequence). The problem of computing the weighted reversal and transposition distance in polynomial time is yet unsolved, but we developed a 1.5 approximation algorithm that works well in practice [4,5], extending the work of Hartman and Sharan for equally weighted reversals and transpositions [6].

Although transpositions really matter and are frequently observed, they were not considered in software tools for whole genome phylogeny due to the computational complications associated with them. The following small example demonstrates the importance of considering transpositions. Table 1 shows the ordering of the mitochondrial genes (starting with the cox1 gene in the circular genome) in *Drosophila melanogaster*, while Table 2 additionally provides the ordering of these genes in *Lithobius forficatus*. Obviously, the mitochondrial genome of *D. melanogaster *can be transformed into that of *Lithobius forficatus *by a transposition of gene 37 and an inverted transposition of gene 2. If one takes solely reversals into account, then the optimal rearrangement scenario would consist of five unnatural reversals.

**Table 1:** Ordering of the mitochondrial genes in Drosophila melanogaster

1	2	3	4	5	6	7	8	9	10
cox1	L2	cox2	K	D	atp8	atp6	cox3	G	nad3
11	12	13	14	15	16	17	18	19	20
A	R	N	S1	E	-F	-nad5	-H	-nad4	-nad4L

21	22	23	24	25	26	27	28	29	30
T	-P	nad6	cob	S2	-nad1	-L1	-rrnL	-V	-rrnS

31	32	33	34	35	36	37	38		
UNK	I	-Q	M	nad2	W	-C	-Y		

**Table 2:** Ordering of the mitochondrial genes in the two organisms.

*D. melanogaster*	1	2	3	4	5	6	7	8	9	10	11	12	13
	14	15	16	17	18	19	20	21	22	23	24	25	26
	27	28	29	30	31	32	33	34	35	36	37	38	
*L. forficatus*	1	3	4	5	6	7	8	9	10	11	12	13	14
	15	16	17	18	19	20	21	22	23	24	25	26	**-2**
	27	28	29	30	31	**37**	32	33	34	35	36	38	

The mitochondrial genome of *Drosophila melanogaster *can be transformed into that of *Lithobius forficatus *by a transposition of gene 37 and an inverted transposition of gene 2.

The multiple genome rearrangement problem was shown to be NP-hard for both the breakpoint and the reversal distance [7,8]. This is true even if one considers only three genomes [7,8]. To put it differently, the breakpoint and the reversal median problem are NP-hard. Recall that in the median problem, we are given a distance or dissimilarity measure *d*, three genomes *g*_1_, *g*_2_, and *g*_3_, and we want to find a genome *M *(a median) such that the sum $\sum_{i=1}^{3}d(M,g_{i})$ is minimized.

### Related work

#### Breakpoint distance

Sankoff and Blanchette [9] solved the multiple genome rearrangement problem for the breakpoint distance by solving the following problem for *each tree topology*: Given a tree topology *T *with the *k *genomes as leaf nodes, assign ancestral genomes to internal nodes of *T *such that the tree is optimal (among all trees with this topology). They proposed to use the breakpoint median problem in an iterative manner to refine rough initial guesses for internal nodes. The breakpoint median problem in turn can be reduced to the traveling salesman problem for which very good heuristics exist. The resulting software BPAnalysis, however, was rather slow. Moret et al. [10] provided a reimplementation of BPAnalysis called GRAPPA that resulted in a speedup by several orders of magnitude.

#### Reversal distance

Moret et al. [11] and Tang et al. [12] showed that using reversal medians instead of breakpoint medians in the method of Sankoff and Blanchette [9] yield better phylogenetic trees, and they further extended the GRAPPA software in this direction. Moreover, Bourque and Pevzner [13] pointed out that the use of the breakpoint distance for generating the tree is disadvantageous because it may result in a tree that is suboptimal under other rearrangement distances. Their own greedy method iteratively builds a phylogenetic tree, based on a heuristic for finding the reversal median (in case of multichromosomal genomes, translocations are taken into account as well). Their method is implemented in the software tool MGR. Wu and Gu devised algorithms that solve an equivalent Steiner tree problem. Besides a nearest path search algorithm on a simple grid [14] (simplifying the grid search algorithm of Sankoff et al. [15]), they also presented a neighbor perturbing algorithm that starts with a minimum spanning tree as a 2-approximation of the optimal Steiner tree and tries to iteratively improve the tree by searching for better Steiner nodes in the neighborhoods of the current nodes [16]. Recently, Bernt et al. [17] presented a software tool called amGRP which further significantly improves upon GRAPPA and MGR.

#### Weighted reversal and transposition distance

To the best of our knowledge, the approach of Cosner et al. [18] is the only one that (indirectly) takes weighted reversals and transpositions into account. In their method, they first use a heuristic to construct a phylogenetic tree according to the breakpoint distance as follows: Genomes are encoded as binary strings, a maximum parsimony technique for binary strings is employed, and then each internal node in the resulting phylogenetic tree is relabeled using BPAnalysis. Then, the weighted reversal and transposition distances on the edges of the tree are heuristically calculated with the software tool DERANGE II [19].

### Our contribution

In this paper, we present a new heuristic algorithm for solving the multiple genome rearrangement problem. Our algorithm is the first one that tries to directly construct an optimal phylogenetic tree under the weighted reversal and transposition distance. We conducted experiments on previously published datasets, and the results are very promising. Because there is no other software tool that can deal with the weighted reversal and transposition distance, we conducted the same experiments with the reversal distance and compared our results with those delivered by other software tools. Although our program was not designed for the reversal distance, the results show that it can compete with state-of-the-art programs in this field.

## Results

### Definitions

In this paper, we consider genomes that consist of a single circular chromosome (like mitochondrial, chloroplast or bacterial genomes). Such genomes are modeled by signed circular permutations. A *signed circular permutation π *= (*π*_1 _... *π*_*n*_) is a permutation of (1...*n*), in which the indices are cyclic (i.e., *n *is followed by 1) and each element is labeled by plus or minus. We will use the term "permutation" as short hand for signed circular permutation. The *reflection *of a permutation *π *is the permutation (-*π*_*n*_... *π*_1_). It is considered to be (biologically) equivalent to *π*. The permutation *id *= (+1 + 2...+*n*) is called the identity permutation. A *segment π*_*i*_... *π*_*j *_(with *j *≤ *i*) of a permutation *π *is a consecutive sequence of elements in *π*, with *π*_*i *_as first element and *π*_*j *_as last element. We consider three possible rearrangement operations on a permutation *π*. A *transposition t*(*i*, *j*, *k*) (with *i *<*j *and *k *<*i *or *k *> *j*) is an operation that cuts the segment *π*_*i *_... *π*_*j*-1 _out of *π*, and reinserts it before the element *π*_*k*_. A *reversal r*(*i*, *j*) (with *i *<*j*) is an operation that reverses the order of the elements of the segment *π*_*i *_... *π*_*j*-1_. Additionally, the sign of every element in the segment is flipped. An *inverted transposition tr*(*i*, *j*, *k*) (with *i *<*j *and *k *<*i *or *k *> *j*) is the composition *t*(*i*, *j*, *k*) ◦ *r*(*i*, *j*) of a reversal and a transposition. In other words, the segment *π*_*i *_... *π*_*j*-1 _will be cut out of *π*, inverted, and reinserted before *π*_*k*_. A *sequence *of rearrangement operations *op*_1_, *op*_2 _,..., *op*_*k *_applied to a permutation *π *yields the permutation $\tilde{\pi }$ = *op*_*k*_(*op*_*k*-1_(...(*op*_1_(*π*))...)). We also say that the sequence is a *sorting sequence of p w.r.t*. $\tilde{\pi }$, as it sorts *π *into $\tilde{\pi }$. A permutation $\hat{\pi }$*lies *on a sorting sequence *op*_1_, *op*_2_,...,*op*_*k *_of *π *if there is a *j *≤ *k *such that *op*_*j*_(*op*_*j*-1_(...*op*_1_(*π*))...)) = $\hat{\pi }$. Each operation *op *is assigned a positive *weight w*(*op*). The weight of a sequence is the sum of the weights of its operations. An *optimal *sorting sequence between *π *and $\tilde{\pi }$ is a sequence of minimum weight transforming *π *into $\tilde{\pi }$. The *weighted genome rearrangement distance *between *π *and $\tilde{\pi }$ (denoted by *d*(*π*, $\tilde{\pi }$)) is the weight of an optimal sorting sequence between them. In this paper, we consider two different genome rearrangement distances, the *reversal distance *and the *weighted reversal and transposition distance*. For the reversal distance, we restrict the set of allowed operations to reversals. Each reversal has the weight 1, i.e. the *reversal distance d*_*r*_(*π*, $\tilde{\pi }$) is the minimum number of reversals required to transform *π *into $\tilde{\pi }$. For the weighted reversal and transposition distance, the weight of an operation only depends on its type:*w*(*op*) = *w*_*r *_if *op *is a reversal, and *w*(*op*) = *w*_*t *_if *op *is a transposition or an inverted transposition. As reversals usually occur much more frequently than transpositions and inverted transpositions, we assume that *w*_*r *_≤ *w*_*t*_. In fact, we assume that *w*_*r *_= 1 ≤ *w*_*t *_≤ 2 because this results in a biologically meaningful balance between reversals and transpositions (see also in Section "Testing, Weight Ratios"). The weighted reversal and transposition distance between two permutations *π *and $\sum_{i=1}^{3}d(M,g_{i})$ is denoted by *d*_*w*_(*π*, $\tilde{\pi }$), and a lower bound for this distance is *d*_*w*_(*π*, $\tilde{\pi }$) ≥ (*w*_*t*_/2)·(*n *- *σ*(*π*, $\tilde{\pi }$)), where *n *is the size of the permutations and *σ*(*π*, $\tilde{\pi }$) is the *score *between the permutations *π *and $\tilde{\pi }$ (for details, see [4]). A *median M*(*π*^1^, *π*^2^, *π*^3^) of three permutations *π*^1^, *π*^2^, and *π*^3 ^is the permutation that minimizes *d*(*M*, *π*^1^) + *d*(*M*, *π*^2^) + *d*(*M*,*π*^3^). Note that in general a median is not unique. An algorithm that solves the median problem (either exactly or heuristically) is called a *median solver*. A *phylogenetic tree *of a set of permutations (genomes) *P *= {*π*^1^,...,*π*^*n*^} is a tree *T *= (*V, E*), where *V *is the set of nodes and *E *is the set of edges of the tree. Each node is labeled by a permutation *v*_*i*_, and there is a bijection between the labels of the leaves and the permutations in *P *(i.e., any element of *P *is label of exactly one leaf). The weight of an edge (*v*_*i*_, *v*_*j*_) is the distance *d*(*v*_*i*_, *v*_*j*_). The weight of a tree *w*(*T*) is the sum of the weights of its edges. The *phylogenetic reconstruction problem *is to find a phylogenetic tree with minimum weight, given the set of permutations *P*.

### Algorithm

The algorithm has two different phases. In the first phase, we use a fast heuristic that creates a tree. This heuristic does not rely on median solvers. In the second phase, this tree will be improved until it converges to a local optimum. We use two different improvement algorithms. One improves the tree topology, while the other improves the labeling of internal nodes by using a median solver. These two algorithms can be run alternatingly until the tree does not improve any further. In practice, the topology of the tree created in the first phase is already very good, so the algorithm for improving the topology has to be run only once. The whole algorithm was designed for the weighted reversal and transposition distance, however, it can also be used for other rearrangement distances as it only requires an algorithm that finds an optimal sorting sequence between two permutations, or at least a good approximation algorithm. Only the second improvement algorithm requires a median solver. We implemented the algorithm for the reversal distance and the weighted reversal and transposition distance. For the reversal distance, there is an efficient exact sorting algorithm [20,21] and a branch-and-bound algorithm for the median that works well in practice [22]. For the weighted reversal and transposition distance, there is a 1.5-approximation for the sorting problem that works well in practice [4,5]. As median solver, we extended Caprara's reversal median solver [22] such that it works on the lower bound for the weighted reversal and transposition distance instead of using the maximum cycle decomposition of the original algorithm. As the algorithm for the weighted reversal and transposition distance is often able to find exact solutions, especially for short distances, the median solver also finds an exact median w.r.t. the weighted reversal and transposition distance in many cases.

#### Creating the tree

We create the tree *T *iteratively, beginning with a tree whose set of nodes consists only of one arbitrary permutation of the set of input permutations *P *= {*π*_1_,...,*π*^*k*^}, i.e. *T*_0 _= (*V*_0_, *E*_0_) = ({*π*}, ∅) with *π *∈ *P*.

In each step, we create the tree *T*_*i *_from the tree *T*_*i*-1 _by choosing a permutation *π *∈ *P*\*V*_*i*-1 _that is not yet a node in the tree, and add this permutation as leaf node. This includes an update of the edge set, and can include the creation of a new internal node. The algorithm terminates when all input permutations are in the tree, i.e., *T *= *T*_*k*_. Choosing the next permutation *π *∈ *P *to be added to the tree *T*_*i*-1_, as well as determining its ancestor node, is done by a heuristic that minimizes the weight of the resulting tree *T*_*i*_. In contrast to previous algorithms, we do not use a median solver for this. Instead, we maintain for each edge (*v*, *v'*) ∈ *E*_*i*-1 _a set of permutations, called the *cloud *of the edge. This cloud can be seen as sets of candidate nodes for internal nodes. For a formal definition of a cloud, we first must define the *δ-vicinity *of an edge.

**Definition 1 ***Let *(*v*, *v'*) *be an edge in a tree and let δ *∈ ℝ. *The δ-vicinity of *(*v*, *v'*) *is defined by*

*Vic*_*δ *_(*v*, *v'*) = {*s *∈ *V *| *d*(*v*, *s*) + *d*(*s*, *v'*) ≤ *d*(*v*, *v'*) + *δ*}

*In the following, we often simply speak of the vicinity of an edge, assuming that δ is fixed*.

Intuitively, this means that the *d*-vicinity of an edge (*v*, *v'*) are all permutations *w *that lie "in between" *v *and *v'*. Splitting the edge into the two edges (*v, w*) and (*w, v'*), and adding the edge (*w*, *π*) will increase the weight of the tree by at most *d*(*w*, *π*) + *δ*. Thus, searching for new internal nodes in the vicinity of edges seems to be a good heuristic. However, even the 0-vicinity of an edge (*v*, *v'*) can be of exponential size w.r.t. *d*(*v*, *v'*). Hence, it is not practicable to search the whole vicinities of the edges, and we have to restrict the search space somehow. In the following, we assume that *d *is a small, fixed number.

**Definition 2 ***The cloud Cloud*(*v*, *v'*) *of an edge *(*v*, *v'*) *in a tree is a subset of Vic*_*δ*_(*v*, *v'*).

Of course, this definition is quite general and does not reflect how to generate "good" clouds. We will present a heuristic for generating clouds in the next section.

**Figure 1:**
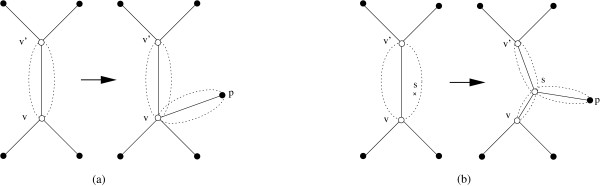
**Adding a node**. A new node *π *can either be added to (a) a node *v *in the tree or (b) to a point *s *in a cloud of an edge (*v*, *v'*). In the latter case, we have to split the edge (*v*, *v'*) into two edges (*v, s*) and (*s, v'*). Clouds are removed/generated accordingly.

Creating the tree *T*_*i *_from *T*_*i*-1 _is now done as follows. We choose an element *π *∈ *P*\*V*_*i*-1 _and either (a) a node *v *∈ *V*_*i*-1 _or (b) an edge (*v*, *v'*) ∈ *E*_*i*-1 _and a permutation *s *∈ *Cloud*(*v*, *v'*), such that the resulting tree *T*_*i *_is of minimum weight. If (a) a node *v *∈ *V*_*i*-1 _is chosen, then the resulting tree is obtained by adding an edge from *π *to *v*, i.e., *V*_*i *_= *V*_*i*-1 _∪ {*π*} and *E*_*i *_= *E*_*i*-1 _∪ {(*v*, *π*)}. If (b) an edge (*v*, *v'*) and a permutation *s *∈ *Cloud*(*v*, *v'*) is chosen, the resulting tree is obtained by replacing (*v*, *v'*) with the two edges (*v, s*) and (*s, v'*) and adding a new edge (*π*, *s*), i.e., *V*_*i *_= *V*_*i*-1 _∪ {*s*, *π*} and *E*_*i *_= *E*_*i*-1 _∪ {(*v*, *s*), (*s*, *v'*), (*π*, *s*)} \ {(*v*, *v'*)}. An illustration can be found in Fig. 1. The weight of *T*_*i *_can be calculated in case (a) by *w*(*T*_*i*_) = *w*(*T*_*i*-1_) + *d*(*v*, *π*) and in case (b) by *w*(*T*_*i*_) = *w*(*T*_*i*-1_) - *d*(*v*, *v'*) + *d*(*v, s*) + *d*(*s, v'*) + *d*(*π*, *s*). It should be pointed out that whenever we add an edge to the tree we also generate its cloud. Analogously, whenever we remove an edge from the tree we also delete its cloud.

The resulting tree does not necessarily fulfill the definition of a phylogenetic tree, as a permutation *π *∈ *P *may correspond to an internal node *v *instead of a leaf node. This can easily be fixed by creating an exact copy *v' *of *v *and adding the edge (*v*, *v'*), i.e. *π *corresponds now to the leaf *v'*.

#### Creating the clouds

The quality and size of the clouds are crucial for the quality of the resulting tree and the running time of the algorithm. Let us consider the two extremes. If the clouds are empty, the algorithm is reduced to Prim's algorithm that finds a minimum spanning tree of *P*. If the cloud of an edge (*v*, *v'*) contains the whole vicinity of the edge, the algorithm will always find a best ancestral genome unless it is not in this vicinity. However, in this case the size of the cloud is exponential w.r.t. *d*(*v*, *v'*). Our goal is to find, for each edge (*v*, *v'*), a cloud of polynomial size w.r.t. *d*(*v*, *v'*) that provides a good coverage of the vicinity of (*v*, *v'*). The main idea of our heuristic is to generate different optimal or near optimal sorting sequences between *v *and *v' *and to select a subset of the permutations that lie on these sorting sequences as the cloud of the edge. To avoid duplicates in the cloud, our algorithm proceeds as follows. First, we define the set *C*_0 _= {*v*}. Then, we iteratively generate the sets *C*_*i *_out of the sets *C*_*i*-1 _by applying to each permutation in *C*_*i*-1 _each operation that decreases the distance to *v'*. Then, we reduce the size of *C*_*i *_by selecting a fixed number of disjoint permutations from *C*_*i*_. As additional heuristic, we select the permutations from *C*_*i *_that minimize the distance to the closest permutation in the set of input permutations *P *that is not yet in the tree. These steps are repeated until we reach the permutation *v'*, i.e. *C*_*m *_= {*v*'}. As cloud, we use the union of the sets *C*_1 _to *C*_*m*-1_.

Note that the resulting cloud is a subset of *Vic*_0_(*v*, *v'*). However, this works only for the reversal distance, as we do not have an exact algorithm for the weighted reversal and transposition distance. In this case, we apply operations that decrease the lower bound instead of decreasing the real distance. Thus, the parameter *δ *depends on the approximation quality of the algorithm for generating the sorting sequence, and we cannot determine it exactly. However, the approximation quality of the algorithm is very good in practice [5], so we can assume that *δ *is small.

#### Improving the topology

**Figure 2:**
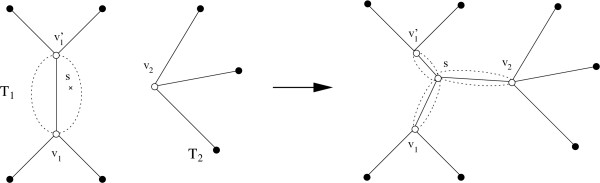
**Improving the tree topology**. The subtrees *T*_1 _and *T*_2 _are reconnected by an edge from *s *to *v*_2_, where *s *∈ *Cloud*(*v*_1_, ${v^{\prime }}_{1}$) and *v*_2 _∈ *V*_2_. The edge (*v*_1_, ${v^{\prime }}_{1}$) must be split into two edges (*v*_1_, *s*) and (*s*, ${v^{\prime }}_{1}$) before the new edge (*s, v*_2_) is added. Clouds are removed/generated accordingly.

The construction phase may get trapped in a local minimum. To avoid this, it is followed by an improvement phase, which iteratively tries to find edges that are better than the existing ones. The input to the improvement algorithm is a tree *T' *= (*V', E'*) for *P *in conjunction with the clouds of the edges. The algorithm works as follows. First, we temporarily remove an edge *e *∈ *E'*. This splits *T' *into two subtrees *T*_1 _= (*V*_1_, *E*_1_) and *T*_2 _= (*V*_2_, *E*_2_). Then we search for a better edge (*w*_1_, *w*_2_) that reconnects these two subtrees as follows. *w*_1 _is either a node in *V*_1 _or a permutation in a cloud of an edge (*v*_1_, ${v^{\prime }}_{1}$) ∈ *E*_1_. In the latter case, we alter the tree *T*_1 _into a tree ${\tilde{T}}_{1}=({\tilde{V}}_{1},{\tilde{E}}_{1})$ by replacing the edge (*v*_1_, ${v^{\prime }}_{1}$) with the two edges (*v*_1_, *w*_1_) and (*w*_1_, ${v^{\prime }}_{1}$), i.e., ${\tilde{V}}_{1}$ = *V*_1 _∪ {*w*_1_} and ${\tilde{E}}_{1}=E_{1}\cup \{(v_{1},w_{1}),(w_{1},{v^{\prime }}_{1})\}\backslash \{(v_{1},{v^{\prime }}_{1})\}$. Otherwise, we set ${\tilde{T}}_{1}$ = *T*_1_. The tree ${\tilde{T}}_{2}$ is defined analogously to ${\tilde{T}}_{1}$ by distinguishing as to whether *w*_2 _is a node in *V*_2 _or a permutation in a cloud of an edge (*v*_2_, ${v^{\prime }}_{1}$) ∈ *E*_2_. The new tree $\bar{T}=(\bar{V},\bar{E})$ is obtained by connecting ${\tilde{T}}_{1}$ and ${\tilde{T}}_{2}$ by the edge (*w*_1_, *w*_2_), i.e., $\bar{V}={\tilde{V}}_{1}\cup {\tilde{V}}_{2}$ and $\bar{E}={\tilde{E}}_{1}\cup {\tilde{E}}_{2}\cup \{(w_{1},w_{2})\}$. For an example, see Fig. 2. If *w*($\bar{T}$) <*w*(*T'*), these changes are accepted (i.e., *T' *:= $\bar{T}$), otherwise they are discarded. When the changes are accepted, we generate the clouds for the new edges and delete the clouds of removed edges.

Note that searching for the edge (*w*_1_, *w*_2_) is done by an exhaustive search for *w*_1 _in all nodes in *V*_1 _and all permutations in the clouds of all edges in *E*_1_, and similarly for *w*_2_. The improvement step is repeated until no further improvement is found.

#### Improving internal nodes

Due to the tree construction algorithm, usually some of the internal nodes are not the median of their neighboring nodes, although they are very close to the median in most cases. The second improvement algorithm further improves the tree by relabeling internal nodes until all internal nodes are the median of their neighbors. For this, we first must ensure that each internal node is of degree 3, since our median solver is designed to find the median of three nodes. Thus, for each internal node *v *with a degree of *k *> 3, we create a node *v' *which is an exact copy of *v*. We reconnect these two nodes such that *v *is connected to *v' *and two of its former neighbors, while *v' *is connected to *v *and all other neighbors of *v*. Thus *v *has now a degree of 3, and *v' *has a degree of *k - *1. We repeat this until all internal nodes have a degree of 3.

Now, for each internal node *v*, we calculate the median *M *of its three neighboring nodes. If the sum of the distances of the neighbor nodes to *M *is less than the sum of the distances of the neighbor nodes to *v*, we replace *v *with *M*. For the reversal distance, we used the exact Caprara median solver, which maximizes the number of cycles in the multiple breakpoint graph and then checks the pairwise distances towards the calculated median (for details, see [22]). For the weighted reversal and transposition distance, we extended Caparara's median solver such that it minimizes the sum of the lower bounds between the calculated median and each of the three input permutations, and then checks the pairwise distances towards the calculated median. Note that there is no efficient exact median solver known for the weighted reversal and transposition distance. However, the median solver finds an exact median w.r.t. the lower bound for the weighted reversal and transposition distance. Thus, we only get inaccuracies by using the approximation algorithm for the weighted reversal and transposition distance, and therefore the approximation ratio of our median solver is as least as good as the approximation ratio of the pairwise distances, which is very good in practice.

The improvement of internal nodes is repeated until the tree does not improve any further, i.e. each node is the median of its neighboring nodes.

### Testing

#### Data sets

We tested our algorithm on the following three biological datasets, which can be considered as benchmarks for phylogenetic reconstruction algorithms based on genome rearrangements.

#### Campanulaceae

This dataset contains 13 chloroplast DNAs of the flowering plant family *Campanulaceae*, where each genome contains 105 markers. It was created by Cosner et al. as test case for their new method MPBE [18], and at that time was ranked among the most challenging datasets for genome rearrangement algorithms.

#### Metazoan

This dataset contains 11 *metazoan *mitochondrial DNAs with 36 different markers. In the context of genome rearrangement algorithms, it was first used in [23]. In the year 2002, Bourque and Pevzner published a tree with 150 reversals, showing that MGR outperforms GRAPPA, as GRAPPA was only able to find a tree with 175 reversals in more than 48 hours [13]. However, GRAPPA has been improved ever since, and the current version is now able to find a tree with 159 reversals in 68 seconds (see Subsection "Experimental results").

#### Protostomes

This dataset contains 62 *protostome *mitochondrial DNAs with 36 different markers. It was first published in [24] and later adjusted in [17] to be used as test scenario for amGRP. The increased amount of data and the larger genome distances make this dataset much more complicated than the metazoan dataset.

### Weight ratios

Two circular genomes (represented by *n *markers) are identical if the number of breakpoints between them is zero and the number of cycles in the breakpoint graph is *n*, respectively (for details, see [25]). Thus, a sequence of rearrangement operations that transforms one genome into the other must reduce the number of breakpoints to zero and increase the number of cycles to *n*, respectively. Because a reversal removes at most two breakpoints and generates at most one new cycle, while a transposition removes at most three breakpoints and generates at most two new cycles, transpositions are generally favored if the weight ratio is 1:1 (*w*_*r *_: *w*_*t*_). On the other hand, if the weight ratio is 1:2 and one uses Eriksen's (1 + ϵ)-approximation algorithm [26], then an optimal sequence of rearrangements will consist almost solely of reversals. In our opinion, a realistic weight ratio for most biological datasets must be somewhere between these two extremes. Exemplarily, we are using the weight ratio 1:1.5.

We performed tests for the weighted reversal and transposition distance with the weight ratios 1:2, 1:1.5, and 1:1. The weight *w*_*r *_for reversals was fixed to 1, while the weight *w*_*t *_for transpositions was set to the corresponding values. We tested our program with different tree improvement strategies: No tree improvement (phylo-n), using only the tree perturbation to improve the topology (phylo-t), using only the median solver to improve the inner nodes (phylo-m), and the combination of both strategies (phylo-tm). Since the result of our algorithm depends on which permutation of the set *P *was chosen as first node, we performed one run for each permutation in *P *as start node.

### Other tools using the reversal distance

As there is no other algorithm that can handle the weighted reversal and transposition distance, we also performed all tests mentioned above with the reversal distance, and compared our results with the results of the following programs.

#### GRAPPA

We used the current version 2.0 [27], which contains some serious improvements above older versions, especially it includes a median solver. The best results were achieved with the parameters -t4 -T4 -n4 -e -m -a -C (for details see the GRAPPA manual). Using DCM-GRAPPA [28] only improved the running times, but usually resulted in worse trees.

#### MGR

We used the current version 2.01. The best results were achieved with the triplet resolution heuristic disabled. Note that the heuristics h3 and h5 are no longer available, thus we could not reproduce some of the results given in [13] and [17].

#### amGRP

We used the version of April 2007. The best results were achieved with the "skewest" heuristic. As amGRP relies on randomness, we performed 50 runs for each data set. Note that this results in a similar number of runs and overall running time for amGRP and our program when using the reversal distance on the protostomes data set. In Subsection "Experimental results", we will only report the result of the best of these runs. For more information about the variance of the output of amGRP, the reader is referred to [17].

All tests were performed on a standard PC (Athlon 64 3200+ 2 GHz CPU, 512 kB L2 Cache, 4 GB RAM, 1000 MHz Bus Speed, Debian Kernel 2.6.22, GCC 4.1.2).

### Comparing the results

While comparing the trees according to the reversal distance is straightforward, one might also be interested in comparing the trees according to the weighted reversal and transposition distance. The only other approach that tackles this task is the one of Cosner et al. [18], however in a very indirect way. First, a phylogenetic tree is generated according to the breakpoint distance, using a maximum parsimony technique for obtaining the tree topology and the software tool BPAnalysis for obtaining the labeling of internal nodes. Then, the weighted reversal and transposition distances of the edges of the tree are heuristically calculated with the software tool DERANGE II [19]. Since BPAnalysis is no longer state-of-the-art and software tools have improved ever since, we investigated whether it is worthwhile to create a tree according to the reversal distance, and then to recalculate the weights of the edges using the weighted reversal and transposition distance. The experiments showed that using DERANGE II or the algorithm devised in [4] led to very similar results, thus we only provide the results of the latter algorithm. To avoid misleading results by using an approximation algorithm, we also recalculated the weights of the edges using the lower bound for the weighted reversal and transposition distance, resulting in a lower bound for the tree weight for a given topology and labeling of internal nodes. To put it differently, recalculating the tree weight with an exact algorithm for the weighted reversal and transposition distance cannot give us a result that is lower than this value. This approach was only feasible for MGR and amGRP because GRAPPA does not report the permutations at internal nodes. One might also ask if using Eriksen's (1 + ϵ)-approximation algorithm [26] would lead to better results for the weight rato 1:2. However, comparing the calculated tree weights with the lower bounds showed that the approximation ratio of our algorithm is very good in practice, especially for this weight ratio, so we did not perform further tests with Eriksen's algorithm.

### Experimental results

#### Weighted reversal and transposition distance

**Figure 3:**
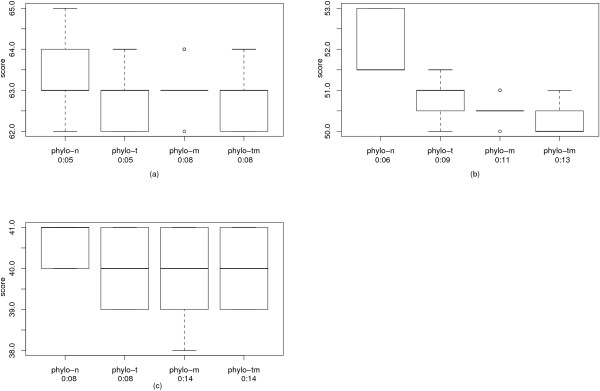
**Variance of the Campanulaceae dataset**. The results of our algorithm for the Campanulaceae dataset over all runs as box plots. For each improvement strategy, also the average running time per run is provided. (a) contains the results for the weight ratio 1:2, (b) for the weight ratio 1:1.5, and (c) for the weight ratio 1:1.

The best results for the Campanulaceae dataset were achieved by phylo-m and phylo-tm, except for the weight ratio 1:1, where phylo-m found a tree with weight 38, while phylo-tm only found a tree with weight 39, see Table 3. A possible reason for this is that the Campanulaceae dataset is easy for the first phase of the algorithm, and the resulting tree has already a very good topology in most cases. As the tree perturbation is a heuristic, a single step can lead to a worse topology. As we can only perform very few improvement steps with this heuristic, there is a risk of having a worse topology than in the beginning. If we have a dataset that allows more improvement steps, the probability of getting a worse tree becomes very small, and we get better trees in almost all cases (see also the results for the protostome dataset). The running time of our algorithm varies from 5 to 14 seconds, depending on the chosen parameters. It has already been mentioned that Cosner et al. [18] constructed a phylogenetic tree for the Campanulaceae dataset according to the breakpoint distance and then relabeled its internal nodes according to the weighted reversal and transposition distance. In this way, they were able to account for transpositions and obtained tree with 40 reversals and 12 transpositions. Note that this is worse than our result. For the weight ratio 1:2, we obtained a tree of weight 62, whereas their tree has weight 64. Our experimental results show that also creating the tree according to the reversal distance and recalculating the edge weights using the weighted reversal and transposition distance is not a competitive approach. Even for the weight ratio 1:2, where reversals are the favored operation, the tree found by our algorithm has a weight of 62, while the trees found by MGR and amGRP have a weight of 63. This gap enlarges for the other weight ratios. Calculating the lower bounds shows that the gap is not caused by inaccuracies during the recalculation of the edge weights but by the approach itself. For details see Table 3. The variance of the different runs of our algorithm is depicted in Fig. 3.

**Table 3:** Results for the Campanulaceae dataset.

	phylo-n	phylo-t	phylo-m	phylo-tm	amGRP	MGR
1:2	62 (62)	62 (62)	62 (62)	62 (62)	63 (63)	63 (63)
	0:05	0:05	0:08	0:08		

1:1.5	51.5 (51)	50 (49.5)	50 (49.5)	50 (49.5)	52.5 (52)	56 (55)
	0:06	0:09	0:11	0:13		

1:1	40 (39)	39 (38)	38 (37)	39 (38)	42 (41)	49 (47)
	0:08	0:08	0:14	0:14		

The results for the Campanulaceae dataset and the weighted reversal and transposition distance. The different lines correspond to the different weight ratio *w*_*r *_: *w*_*t*_, while the columns correspond to the different improvement strategies. Each entry consists of the weight of the best tree, the lower bound for the weight of the tree in brackets (with the given topology and labeling of internal nodes), and the average running time per run in minutes. The results of MGR and amGRP were achieved by creating a tree w.r.t. the reversal distance, and recalculating the tree weight with the weighted reversal and transposition distance. The running time for this method is mainly the running time of amGRP or MGR (see Table 6), thus we did not provide explicit running times for these results.

**Figure 4:**
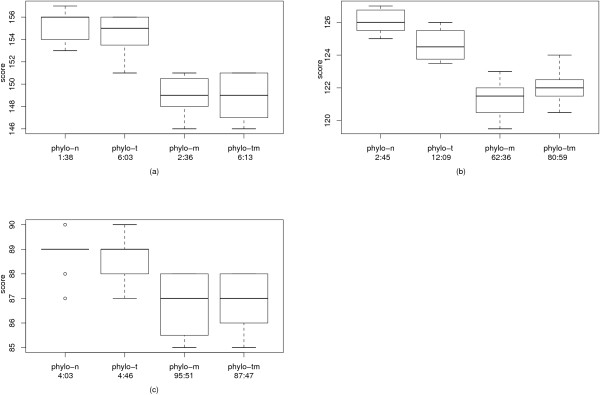
**Variance of the metazoan dataset**. The results of our algorithm for the metazoan dataset over all runs as box plots. For each improvement strategy, also the average running time per run is provided. (a) contains the results for the weight ratio 1:2, (b) for the weight ratio 1:1.5, and (c) for the weight ratio 1:1.

The best results for the metazoan dataset were achieved by phylo-m and phylo-tm, except for the weight ratio 1:1.5, where phylo-m found a tree with weight 119.5, while phylo-tm only found a tree with weight 120.5. While the algorithm was very fast for the weight ratio 1:2 (2:36 min per run for phylo-m and 6:13 for phylo-tm), the running times increased for the other weight ratios when the median solver was used. Nevertheless, the running times of about 1.5 hours per run are still feasible in practice. A comparison of our trees with those found by amGRP and MGR again showed that the direct usage of the weighted reversal and transposition distance is of advantage. Except for the weight ratio 1:2, for which amGRP found a better tree, the trees found by our program have a lower weight under the weighted reversal and transposition distance than the trees found by the other programs. Details can be found in Table 4. The variance of the different runs of our algorithm is depicted in Fig. 4.

**Table 4:** Results for the metazoan dataset.

	phylo-n	phylo-t	phylo-m	phylo-tm	amGRP	MGR
1:2	153 (153)	151 (151)	146 (145)	146 (146)	142 (141)	151 (151)
	1:38	6:03	2:36	6:13		

1:1.5	125 (125)	123.5 (123.5)	119.5 (119.5)	120.5 (120.5)	125.5 (123.5)	134.5 (129)
	2:45	12:09	62:36	80:59		

1:1	87 (87)	87 (87)	85 (85)	85 (85)	110 (106)	116 (107)
	4:03	4:46	95:51	87:47		

The results for the metazoan dataset and the weighted reversal and transposition distance.

**Figure 5:**
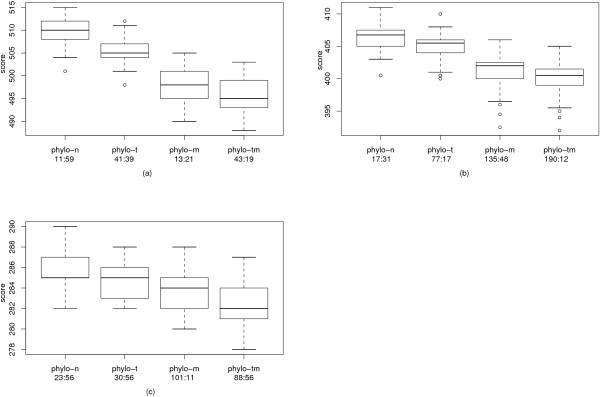
**Variance of the protostomes dataset**. The results of our algorithm for the protostomes dataset over all runs as box plots. For each improvement strategy, also the average running time per run is provided. (a) contains the results for the weight ratio 1:2, (b) for the weight ratio 1:1.5, and (c) for the weight ratio 1:1.

The best results for the protostomes dataset were achieved by phylo-tm, improving the tree topology paid off for all weight ratios. Again, the algorithm is fast for a weight ratio of 1:2 (43:19 min), and the running times increase for the other weight ratios when the median solver is used. Still, the running times of up to about 3 hours per run are feasible in practice. Again, the trees found by our program have a lower weight under the weighted reversal and transposition distance than the trees found by amGRP and MGR. For details we refer to Table 5. The variance of the different runs of our algorithm is depicted in Fig. 5.

**Table 5:** Results for the protostomes dataset.

	phylo-n	phylo-t	phylo-m	phylo-pm	amGRP	MGR
1:2	501 (497)	498 (494)	490 (487)	488 (485)	496 (490)	522 (520)
	11:59	41:39	13:21	43:19		

1:1.5	400.5 (399)	400 (398.5)	392.5 (392.5)	392 (392)	431.5 (418)	446.5 (441.5)
	17:31	77:17	135:48	190:12		

1:1	23:56	30:56	101:11	88:56	365 (347)	388 (363)
	23:56	30:56	101:11	88:56		

The results for the protostomes dataset and the weighted reversal and transposition distance.

#### Reversal distance

Although our algorithm was not particularly designed for this distance measure, we also performed all tests with the reversal distance to compare our program with the other programs. The best result for the Campanulaceae dataset was achieved by our program and GRAPPA (64 reversals). However, GRAPPA needed over 20 minutes to find a tree, while our program needed only a few seconds per run. The best result for the metazoan dataset was achieved by amGRP (143 reversals in 35 seconds), our own program was only slightly worse (144 reversals in 49 seconds). GRAPPA (159 reversals in 1:08 min) and MGR (151 reversals in 36:42 min) were both outperformed. Also for the protostomes dataset, the best result was achieved by amGRP (501 reversals in 6:52 min). The tree found by our program was slightly worse, but it was faster than amGRP for this dataset (505 reversals in 4:41 min). Again, MGR was outperformed (528 reversals in about 20 hours). GRAPPA was not able to find any solution at all within five days. A summary of these results is shown in Table 6. The tremendous improvement in speed of our program versus the weighted reversal and transposition distance can be explained by the fact that calculating the reversal distance can be done in linear time [2], while we use a cubic algorithm for the weighted reversal and transposition distance.

**Table 6:** Results for the reversal distance.

	phylo-tm	amGRP	MGR	GRAPPA
Campanulaceae	64	65	66	64
	0:03	0:01	0:47	20:29

Metazoan	144	143	151	159
	0:49	0:35	36:42	1:08

Protostomes	505	501	528	n.a.
	4:41	6:52	1248:49	

The results for the datasets when using the reversal distance. Only the results of the best strategies are shown. GRAPPA did not find any solution for the protostome dataset within 5 days.

## Conclusion

Our algorithm achieves two improvements over previous works on this topic. First, it does not rely on a specific genome rearrangement distance. It can be used for any genome rearrangement distance as long as there is at least a good approximation algorithm for generating a sorting sequence between two genomes. Second, it avoids median solvers as long as possible. The median problem is NP-hard for the breakpoint distance and the reversal distance, and most likely also for the other genome rearrangement distances. Although solving the median problem may be feasible for small problems, it is getting more and more complicated for larger genomes. Thus, in our opinion, median solvers should be avoided as long as possible. We have shown that our algorithm can compete with the currently best algorithms w.r.t. the reversal distance. To the best of our knowledge, our algorithm is currently the only one that directly tackles the phylogenetic reconstruction problem for the weighted reversal and transposition distance.

We implemented the algorithm in *C*++. The program is open source and can be obtained from the authors.

## Authors' contributions

The initial idea of the algorithm was developed by all three authors. MB developed and tested the software.
